# Safety and tolerability of intradiscal implantation of combined autologous adipose-derived mesenchymal stem cells and hyaluronic acid in patients with chronic discogenic low back pain: 1-year follow-up of a phase I study

**DOI:** 10.1186/s13287-017-0710-3

**Published:** 2017-11-15

**Authors:** Hemant Kumar, Doo-Hoe Ha, Eun-Jong Lee, Jun Hee Park, Jeong Hyun Shim, Tae-Keun Ahn, Kyoung-Tae Kim, Alexander E. Ropper, Seil Sohn, Chung-Hun Kim, Devang Kashyap Thakor, Soo-Hong Lee, In-Bo Han

**Affiliations:** 1 Department of Neurosurgery, CHA University, CHA Bundang Medical Center, Seongnam-si, Gyeonggi-do 13496 South Korea; 2 Department of Radiology, CHA University, CHA Bundang Medical Center, Seongnam-si, Gyeonggi-do 13496 South Korea; 3 CHA Biotec®, Seongnam-si, Gyeonggi-do 13488 South Korea; 4 Department of Neurosurgery, Shim Jeong Hospital, Seoul, 151715 South Korea; 5 Department of Orthopedic Surgery, CHA University, CHA Bundang Medical Center, Seongnam-si, Gyeonggi-do 13496 South Korea; 6 Department of Neurosurgery, Kyungpook National University Hospital 130, Dongdeok-ro, Jung-gu, Daegu, 41944 Korea; 7 Department of Neurosurgery, Baylor College of Medicine, Houston, TX 77030 USA; 8 Department of Plastic and Reconstructive Surgery, CHA University, CHA Bundang Medical Center, Seongnam-si, Gyeonggi-do 13496 South Korea; 9 Anioplex LLC, Campbell, CA 95008 USA; 10 Department of Biomedical Science, CHA University, Seongnam-si, Gyeonggi-do 13496 South Korea

**Keywords:** Intervertebral disc degeneration, Adipose-derived mesenchymal stem cells, Nucleus pulposus, Cell therapy, Hyaluronic acid

## Abstract

**Background:**

Adipose tissue-derived mesenchymal stem cells (AT-MSCs) offer potential as a therapeutic option for chronic discogenic low back pain (LBP) because of their immunomodulatory functions and capacity for cartilage differentiation. The goal of this study was to assess the safety and tolerability of a single intradiscal implantation of combined AT-MSCs and hyaluronic acid (HA) derivative in patients with chronic discogenic LBP.

**Methods:**

We performed a single-arm phase I clinical trial with a 12-month follow-up and enrolled 10 eligible chronic LBP patients. Chronic LBP had lasted for more than 3 months with a minimum intensity of 4/10 on a visual analogue scale (VAS) and disability level ≥ 30% on the Oswestry Disability Index (ODI). The 10 patients underwent a single intradiscal injection of combined HA derivative and AT-MSCs at a dose of 2 × 10^7^ cells/disc (*n* = 5) or 4 × 10^7^ cells/disc (*n* = 5). Safety and treatment outcomes were evaluated by assessing VAS, ODI, Short Form-36 (SF-36), and imaging (lumbar spine X-ray imaging and MRI) at regular intervals over 1 year.

**Results:**

No patients were lost at any point during the 1-year clinical study. We observed no procedure or stem cell-related adverse events or serious adverse events during the 1-year follow-up period. VAS, ODI, and SF-36 scores significantly improved in both groups receiving both low (cases 2, 4, and 5) and high (cases 7, 8, and 9) cell doses, and did not differ significantly between the two groups. Among six patients who achieved significant improvement in VAS, ODI, and SF-36, three patients (cases 4, 8, and 9) were determined to have increased water content based on an increased apparent diffusion coefficient on diffusion MRI.

**Conclusions:**

Combined implantation of AT-MSCs and HA derivative in chronic discogenic LBP is safe and tolerable. However, the efficacy of combined AT-MSCs and HA should be investigated in a randomized controlled trial in a larger population.

**Trial registration:**

ClinicalTrials.gov NCT02338271. Registered 7 January 2015.

## Background

Chronic low back pain (LBP) is one of the leading causes of disability and has an enormous social and economic impact on patients and their family members [1, 2]. The actual cost of back pain is difficult to estimate because it is affected by many factors, but has been estimated to be as high as US$500 billion [2, 3]. Approximately 80% of adults experience LBP at some point in their lifetime, with a prevalence ranging from 15 to 45%, and the prevalence of chronic LBP increases with age due to global population aging, changes in lifestyle, and occupational stresses [2, 4, 5].

Intervertebral disc (IVD) degeneration is characterized by progressive and irreversible IVD degradation due to many different causes [2, 4, 5]. Although not all patients with radiological evidence of IVD degeneration have LBP, IVD degeneration is considered one of the major causes of chronic LBP [6, 7]. The causes of IVD degeneration are complex and multifactorial, and include genetic, nutritional, and mechanical influences [2, 8]. IVD degeneration is characterized by progressive decline in nucleus pulposus (NP) hydration due to the loss of extracellular matrix (ECM) molecules such as aggrecan and collagen [2, 8]. This decreased disc hydration results in a loss of mechanical tension in the collagen fibers of the annulus fibrosus and results in abnormal spinal axial loading forces and segmental instability [9]. Eventually, IVD degeneration can result in abnormalities of other parts of the IVD such as the endplate and facet joint and develop into serious conditions, such as disc herniation, spondylolisthesis, spinal canal stenosis, or facet joint syndrome [10, 11]. Repair or arrest of disc degeneration in the early stages might prevent these more serious sequelae.

The existing surgical procedures to correct herniated disc or spinal stenosis caused by IVD degeneration do not address the abnormal increase in proinflammatory cytokine levels of the degenerated disc or the inherent loss of functional native cells within the IVD. Current research is focusing on the development of stem cell-based therapies to inhibit aberrant cytokine production, stimulate matrix anabolism, and repopulate and influence the native cells. Several adult stem cell types have been applied in preclinical and clinical studies [2, 12–14], and mesenchymal stem cells (MSCs) have been anticipated as an ideal cell source for IVD regeneration because of their immunomodulatory functions and capacity for cartilage differentiation. A small human clinical trial demonstrated improved pain and disability scores as well as increased water content in the disc 12 months after MSC implantation [12–14]. In previous preclinical studies, implanted MSCs enhanced ECM production, particularly glycosaminoglycan synthesis, and increased disc height and hydration [15–20]. Additionally, MSCs isolated from bone marrow and adipose tissue are able to differentiate into an NP-like phenotype [20–25]. However, MSC implantation in degenerated IVD may induce osteophyte formation by cell leakage. Combined implantation of MSCs engrafted in cell carriers such as hyaluronic acid (HA) and fibrin has been suggested to reduce this risk of osteophyte formation [26, 27].

To our knowledge, the safety and tolerability of combined implantation of autologous adipose tissue-derived MSCs (AT-MSCs) and HA derivative has not been tested clinically in patients with chronic discogenic LBP. Therefore, the purpose of this phase I clinical trial was to investigate the safety and tolerability of a single intradiscal implantation of combined AT-MSCs and HA derivative in patients with chronic discogenic LBP.

## Methods

### Study design

The study protocol was approved by the institutional review boards and ethics committees of CHA Bundang Medical Center (BD2013-158, December 2013). The study was also approved by the Ministry of Food and Drug Safety of South Korea (MFDS, 1403-6583-4849-0120, June 2014), and was conducted in accordance with good clinical practice guidelines (ISO 14155) and the Declaration of Helsinki. The study is registered in an internationally recognized clinical trials database (ClinicalTrials.gov NCT02338271). Written informed consent was obtained from all study subjects.

We performed a single-arm, open-label, phase I clinical trial at one trial center (CHA Bundang Medical Center) with a 12-month follow-up period. We investigated safety issues and treatment outcomes for combined implantation of AT-MSCs and HA derivative using a visual analogue scale (VAS), the Oswestry Disability Index (ODI), Short Form-36 (SF-36), and imaging techniques such as lumbar spine X-ray imaging and magnetic resonance imaging (MRI) in patients with chronic discogenic LBP refractory to conventional treatments. Table 1 presents the study assessment schedule. In total, 15 chronic LBP patients were screened for enrollment in the trial. We had diagnosed all of these patients according to clinical and neuroimaging evidence [28, 29]. From these potential participants, we recruited 11 eligible chronic LBP patients. The study enrolled patients from April 2015 to September 2016.

**Table 1:** Assessment schedule

	SV	V1	V2	V3	V4	V5	V6	V7	V8	V9
Day	–35	–21	0	1	7	30	90	180	270	360
Time window (days)	NA	±3	±1	±1	±3	±7	±7	±7	±7	±7
Informed consent	×									
Physical examination	×	×	×	×	×	×	×	×	×	×
Vital signs	×	×	×	×	×	×	×	×	×	×
Medical history	×									
Laboratory assessments	×		×	×	×	×	×	×	×	×
VAS	×	×	×	×	×	×	×	×	×	×
ODI	×	×	×	×	×	×	×	×	×	×
SF-36	×	×	×	×	×	×	×	×	×	×
Lumbar spine X-ray imaging	×					×		×		×
Lumbar spine MRI	×					×		×		×
BMD measurement	×									
Electromyography	×									
Discography	×									
Liposuction		×								
Stem cell transplantation			×							
AE assessment					×	×	×	×	×	×
SAE assessment					×	×	×	×	×	×

*SV* screen visit, *NA* not applicable, *V* visit, *VAS* visual analogue scale, *ODI* Oswestry Disability Index, *SF-36* Short Form-36, *MRI* magnetic resonance imaging, *BMD* bone mineral density, *AE* adverse events, *SAE* severe adverse event

We evaluated both the safety and tolerability of combined implantation of AT-MSCs and HA derivative according to physical and neurological examination, adverse event (AE) assessments, and changes in laboratory parameters. Measures of potential efficacy included changes in the VAS, ODI, and SF-36 scores self-reported by patients and changes in disc water content determined by MRI. We scheduled patient follow-up visits for clinical, laboratory, and imaging assessments at specified intervals of 1 week and 1, 3, 6, 9, and 12 months after combined implantation of AT-MSCs and HA derivative.

### Enrollment criteria

Inclusion criteria for enrollment in the clinical study were: both sexes; age between 19 and 70 years; symptoms of axial chronic discogenic LBP for at least 3 months, with a minimum intensity of 4/10 on the VAS; disability level ≥ 30% on the ODI; failure to respond to conventional treatments including medication, intensive physical rehabilitation, and local anesthetic infiltration in facet joints or medial branches; moderate grade of IVD degeneration (Pfirrmann’s grade III–IV at one or two levels based on T2-weighted MRI) [29]; and degenerative symptomatic discs confirmed by discography. Discography was used to identify the specific symptomatic degenerated disc. Exclusion criteria consisted of: pregnancy or breastfeeding; previous history of surgery of the lumbo-sacral area; severe herniated disc or stenosis requiring surgery; Modic type 3 change; evidence of spinal infection on MRI; disc space collapse > 50%; uncontrolled hypertension despite receiving optimal medication; uncontrolled diabetes despite receiving optimal medication; other serious systemic diseases such as cancer, autoimmune disease, blood disease, kidney disease, and liver disease; and allergies to HA.

### Discography

All patients underwent discography to determine whether the degenerated disc was the cause of chronic LBP. Discography was performed under C-arm fluoroscopy with a 25-gauge spinal needle, using a standard posterolateral approach by one spine surgeon (I-BH). Once the needle was correctly positioned in the center of the disc, nonionic contrast medium (Visipaque™, iodixanol) with 6 mg/ml cephalosporin was injected slowly into the NP of each degenerative disc at L3–4 to L5–S1, based on T2-weighted sagittal MRI, under low pressure controlled by hand. Positive discography was defined when the patient experienced an exact reproduction of the usual pain after injection of contrast medium. Computed tomography (CT) was performed after discography to demonstrate intradiscal clefts and radial tears.

### Primary and secondary endpoints

The primary endpoints were the safety and tolerability of combined implantation of AT-MSCs and HA derivative in patients with chronic LBP. Systemic monitoring included physical and neurological examinations, monitoring of vital signs, and peripheral blood testing. We recorded AEs and serious adverse events (SAEs) attributable to the treatment that patients received during treatment and follow-up. The secondary endpoints consisted of changes in VAS, ODI, SF-36, and disc water content from baseline to 12 months after combined implantation of AT-MSCs and HA derivative. Clinical evaluations and laboratory checks were performed at 1 week and 1, 3, 6, 9, and 12 months after combined implantation of AT-MSCs and HA derivative. Initial and follow-up lumbar spine X-ray imaging and MRI (apparent diffusion coefficient (ADC) mapping from diffusion-weighted imaging (DWI)) were performed prior to cell transplantation and at 1 month, 6 months, and 1 year after transplantation to determine changes in disc height and water content in the treated discs.

### MRI acquisition

The MRI images of the lumbar spine in each subject were obtained using a 1.5-T MR scanner (Signa HDxt; GE Medical Systems, Milwaukee, WI, USA). For evaluation of IVD degeneration, T2-weighted imaging in the sagittal plane and DWI in the axial plane were performed. T2-weighted imaging parameters were as follows: repetition time (TR), 3200 msec; echo time (TE), 121.3 msec; number of excitations (NEX), 2; slice thickness, 3 mm; and interslice gap, 0.33 mm. DWI was performed in the axial plane from the L3 level to the S2 level with spin-echo planar imaging sequences using a *b* value of 500 s/mm^2^, and other parameters were as follows: TR, 13,000 msec; TE, 65.3 msec; TI (inversion time), 180 msec; slice thickness, 3.5 mm; matrix number, 96 × 128; NEX, 12.

**Fig. 1:**
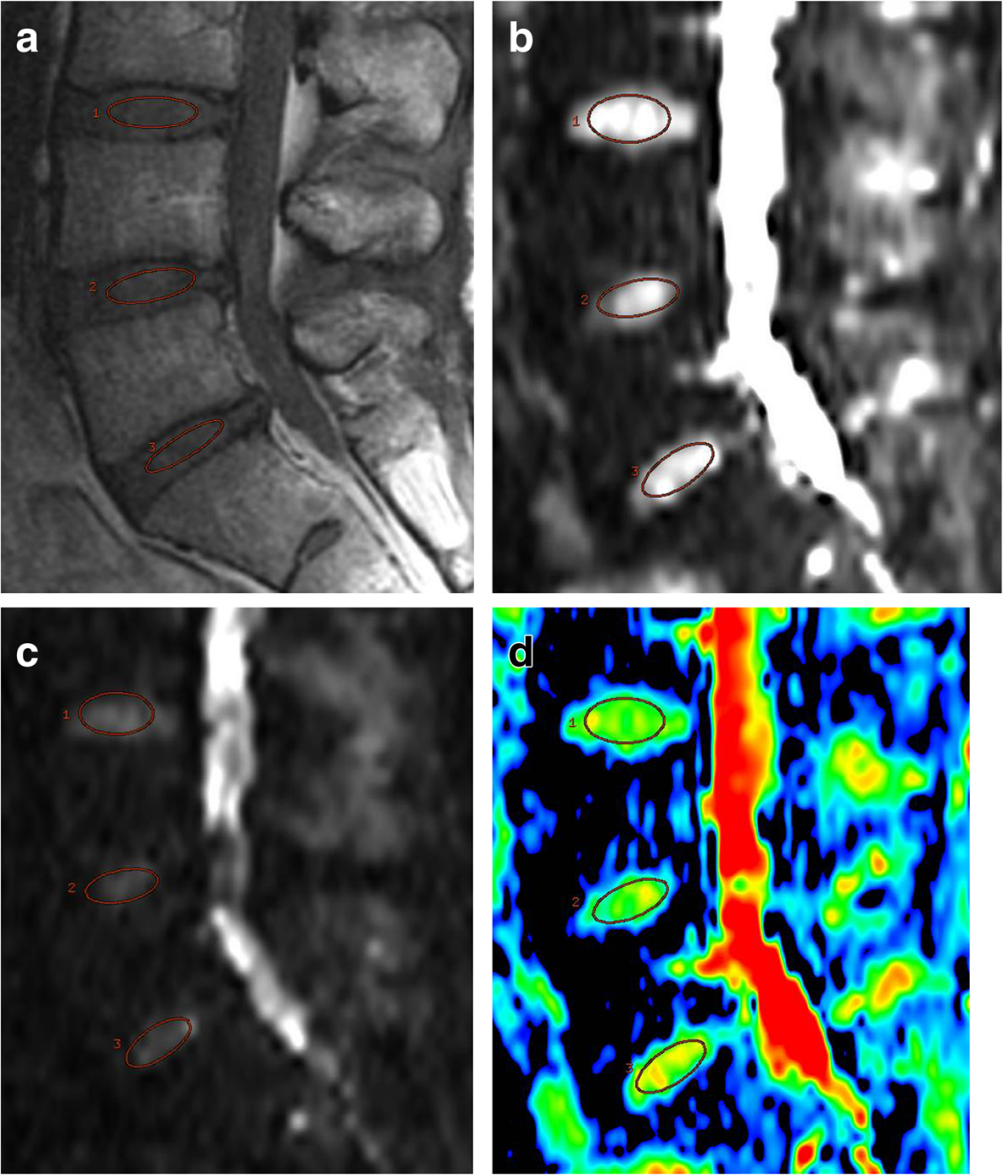
Measurement of the apparent diffusion coefficient (ADC) in lumbar degenerated discs. Mid-sagittal T2-weighted images were chosen and a region of interest (ROI, 50 mm^2^) placed in the central half of each disc. Sagittal fast spin echo (FSE) T2-weighted images (**a**) and diffusion-weighted images (DWI) with *b* factors of 0 (**b**) and 500 s/mm^2^ (**c**). ROI in the T2-weighted image copied to the ADC map from the DWI at the same level (**d**)

To provide a quantitative biomarker for IVD condition, DWI data analysis was performed in an imaging workstation (Advanced Workstation; GE Medical Systems). DWI visualizes diffusion of water molecules directly and ADC can be derived from a series of measurements with different diffusion sensitivity (*b* values) [30]. The ADC of the IVD was obtained from sagittal reformatted DWI images. An experienced radiologist (D-HH) evaluated IVD degeneration and quantitatively analyzed the images without knowledge of the clinical information. IVD degeneration was subjectively graded using Pfirrmann’s classification [29, 31]. The water content of the IVD was evaluated using ADC maps, which were generated from the series of DWI images. To quantify diffusions in the IVD, the ellipsoid region of interest (ROI, 50 mm^2^) was placed in the central half of the IVD. An elliptical ROI, on the middle section of sagittal fast spin echo (FSE) T2-weighted images (Fig. 1a), was manually drawn in the inner portion of each lumbar disc, indicating the regions of the NP. The ROIs were then copied to the ADC map (DWI with *b* factors of 0 (Fig. 1b) and 500 s/mm^2^ (Fig. 1c)) at the same level, and the ADC values (Fig. 1d) of the NP were calculated for analysis.

### Isolation and expansion of autologous AD-MSCs

Subcutaneous abdominal adipose tissue was harvested via liposuction performed 3 weeks before transplantation by a plastic surgeon (C-HK) in the operating room under local anesthesia. In total, 150 ml of tissue was obtained by suction using 50-ml syringes. The patient was discharged after a 4-h observation period. The liposuction sample was transported to the good manufacturing practice (GMP) facility of CHA Biotech Co., Ltd (Pangyo, Seongnam, South Korea). The lipoaspirate was processed in a sterile environment using strict aseptic techniques. The adipose tissues were washed twice with Dulbecco’s phosphate-buffered saline (DPBS) with Ca^2+^ and Mg^2+^, and AT-MSCs were isolated by enzymatic dissociation of adipose tissue followed by centrifugation at room temperature. The cells were plated on flasks and incubated at 37 °C in a humidified incubator under a 5% CO_2_ atmosphere. The medium was then changed every 2–3 days until the cells achieved 80–90% confluence. AT-MSCs at passages 1, 3, 6, and 12 were used for cell characterization. The cells were characterized by flow cytometry (fluorescence-activated cell sorting (FACS)) using surface markers for MSCs: CD44, CD73, and CD29 as positive markers and CD45 as a negative surface marker. The cells were suspended at a concentration of 5 × 10^7^ cells/1.25 ml of normal saline/vial. Safety was established through quality control of the final product based on analysis of genetic stability; microbiological, mycoplasmal, and endotoxin contamination; purity; and cell viability. For each patient, cultured AT-MSCs at passage 3 were delivered to the institute’s operating room in a cold box at a temperature of approximately 4 °C.

### Preparation of HA derivative (Tissuefill®) for cell delivery

Tissuefill® (HA derivative; CHA Meditech Co., Ltd, Daejeon, South Korea) is a clear, transparent, and viscoelastic gel composed of a HA derivative of nonanimal origin. The gel is cross-linked with butanediol diglycidyl ether, and is resorbed almost completely in the body via enzymatic reactions. This HA derivative has been approved as a material for cell delivery and filling of tissue defects by South Korea’s MFDS. Tissuefill® was purchased from CHA Meditech Co., Ltd to investigate the safety and tolerability of combined use of autologous AT-MSCs and HA derivative. In our previous study [32], we determined the optimal concentration of HA derivative for injection into the degenerated disc and found no cytotoxicity in vitro.

### Transplantation of AT-MSCs in combination with HA

After confirmation of IVD degeneration using T2-weighted MRI, symptomatic discs were chosen for stem cell transplantation based on discographic findings. The method for this procedure was similar to that of discography. With the patient in a prone position, the injection site was treated with local anesthetic (1% buffered lidocaine). The cell product (5 × 10^7^ cells/1.25 ml of normal saline/vial) and HA derivative (Tissuefill®) were delivered to the operating room from the GMP facility. We implanted AT-MSCs in combination with HA derivative (1% Tissuefill®) into the center of the symptomatic disc via a percutaneous 22-gauge spinal needle. The safety and tolerability of combined implantation of autologous AT-MSCs and HA derivative were evaluated in two dose-escalation cohorts of 2.0 × 10^7^ and 4.0 × 10^7^ AT-MSCs/disc. The cohorts were enrolled sequentially. The first five consecutive subjects received a mixture of 0.5 ml of stem cell suspension (2 × 10^7^ cells/disc), 0.5 ml of normal saline, and 1 ml of Tissuefill® (1%), and the second five consecutive subjects received a mixture of 1.0 ml of stem cell suspension (4 × 10^7^ cells/disc) and 1 ml of Tissuefill® (1%). In all subjects, a total volume of 2 ml was injected into the center of the symptomatic disc under the guidance of a C-arm fluoroscope. The spinal needle was left in place for 5 minutes to prevent leakage of cells and HA derivative. Patients were prescribed pain medicine to be used as needed for 3 days and put on restricted physical activity for 2 weeks.

### Statistical analysis

The researcher performing the data analysis was blinded to the group allocation. The primary focus of the data analysis was to determine any treatment effect (with 95% confidence intervals) at each follow-up point (1 week and 1, 3, 6, 9, and 12 months post transplantation). In addition, we evaluated intergroup (2 × 10^7^ cells/disc vs 4 × 10^7^ cells/disc) treatment effect differences at each follow-up point (1 week and 1, 3, 6, 9, and 12 months post transplantation). The Wilcoxon signed-rank test and paired *t* test were used to compare pretreatment and post-treatment values (VAS, ODI, SF-36, and ADC values). A linear mixed model was applied to compare the effect of stem cells between the two groups (2 × 10^7^ cells/disc vs 4 × 10^7^ cells/disc). The data are presented as the mean ± standard deviation. Results were regarded as significant when *P* < 0.05. Differences between patients were assessed using ANOVA and post-hoc testing at each time point. SPSS V software was used to conduct the analysis.

## Results

### Patient characteristics

Out of 11 enrolled subjects, one subject withdrew consent after isolation of subcutaneous adipose tissue, and 10 subjects completed the trial. All patients remained in the trial for the 12-month follow-up period. Table 2 presents the patients’ characteristics and demographic data at the time of enrollment. There were six male and four female patients, and their age ranged from 30 to 64 years with a mean age of 43.5 years. Chronic LBP symptom durations ranged from 7 to 96 months (mean 48.3 months). Six patients (cases 2, 4, 5, 7, 9, and 10) had a normal body mass index (BMI; healthy BMI 18.5–24.9), three patients (cases 1, 3, and 8) were overweight (BMI 25–29.9), and one patient (case 6) was obese (BMI 30 or higher). Based on discographic findings, AT-MSCs combined with HA derivative were implanted into the L4/5 disc in nine patients. Only case 6 received stem cells in the L4/5 and L5/S1 discs based on discographic findings.

**Table 2:** Patient characteristics and characterization of cell products

		Patient number									
		1	2	3	4	5	6	7	8	9	10
Sex (M/F)		F	F	F	M	M	M	F	M	M	M
Age (years)		37	42	49	42	44	41	30	32	64	54
BMI (kg/m^2^)		27.9	22.6	26.5	22.2	20.3	38	20.2	26.7	23.1	23.5
Hypertension (yes/no)		N	N	Y	N	N	Y	N	N	N	N
Diabetes mellitus (yes/no)		N	N	N	N	N	N	N	N	N	N
Smoking history (yes/no)		N	N	N	N	N	Y	N	N	Y	N
Duration of LBP (months)		96	12	14	29	7	37	96	72	36	84
Implanted disc level		L4/5	L4/5	L4/5	L4/5	L4/5	L4/5, L5/S1	L4/5	L4/5	L4/5	L4/5
Preoperative VAS		8	7	6	7	4	7	6	6	6	7
Preoperative ODI		40	34	30	50	32	72	54	32	32	60
Preoperative Pfirrmann’s grade		IV	IV	IV	IV	IV	IV	IV	IV	IV	IV
Cell number (× 10^7^/vial)		4.7	5.3	5.4	5.3	5.4	5.0	5.3	5.0	5.2	4.7
Cell viability (%)		93.62	91.55	97.57	88.30	97.09	89.44	87.13	94.38	94.55	89.93
Cell surface marker	CD44 (%)	94.50	84.80	98.00	99.90	99.90	100	99.8	99.9	98.3	99.2
	CD73 (%)	98.40	98.60	99.90	100.00	99.60	100	100	100	99.9	99.9
	CD45 (%)	1.00	0.81	0.20	0.48	0.51	0.75	0.19	0.49	0.7	0.67
	CD29 (%)	90.80	97.50	99.80	99.90	99.90	100.00	100.00	100.00	100.00	99.90
	TGF-β receptor III	98.20	99.10	99.60	100.00	99.70	99.10	99.90	100.00	99.40	99.80

*M* male, *F* female, *BMI* body mass index, *LBP* low back pain, *VAS* visual analogue scale, *ODI* Oswestry Disability Index, *TGF-β* transforming growth factor beta

### Characteristics of AT-MSCs

Autologous MSCs were isolated from subcutaneous abdominal adipose tissue and cultured for 3 weeks. The number of cells per vial from each patient ranged from 4.7 × 10^7^ to 5.4 × 10^7^ cells and the cell viability ranged from 87.13 to 97.57% (Table 2). The AT-MSCs displayed a typical fibroblast-like, spindle-shaped morphology. AT-MSCs at passage 3 exhibited high expression of the mesenchymal surface markers CD73, CD44, and CD29, which were expressed in > 90% of the total cell population. In contrast, only a small proportion (<1%) of AT-MSCs expressed the hematopoietic marker CD45. More than 98% of the AD-MSCs from each patient were positive for the surface marker, type III TGF-β receptor (TβRIII) (Table 2). Testing for 15 different types of viruses in the AT-MSCS was performed at the end of the culture period and no evidence of viral infection was found. AT-MSCs were also negative for aerobic and anaerobic bacteria as well as mycoplasma in all cases. The endotoxin levels in the culture supernatant of the AD-MSCs were normal. After 3 weeks of culture of autologous AT-MSCs, the passage 3 AT-MSCs in HA derivative were transplanted percutaneously.

### Primary endpoints: safety and tolerability

We discharged all patients 4 h after cell transplantation. During the treatment and 12-month follow-up period, we did not observe any AEs or SAEs related to the cell transplantation. Blood tests showed no significant findings for laboratory parameters, which were evaluated for safety issues after 1 week and 1, 3, 6, 9, and 12 months in comparison to the baseline.

### Secondary endpoints

Secondary endpoints included improvement from baseline in VAS, ODI, and SF-36 scores. The clinical success rate was determined as the percent change in VAS and ODI scores between pretreatment and 12-month mean follow-up; the treatment was defined as successful when patients reported pain reduction ≥ 50% and ODI improvement ≥ 50% compared with pretreatment VAS and ODI scores.

**Fig. 2:**
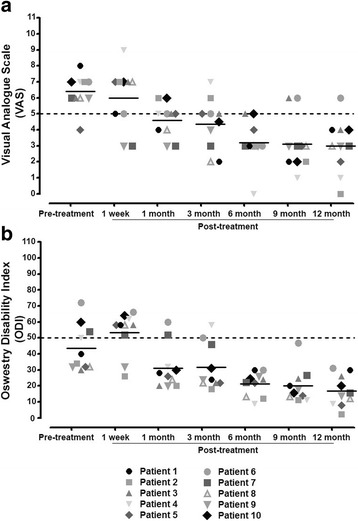
Visual analogue scale (**a**) and Oswestry Disability Index (**b**). Six patients (cases 2, 4, 5, 7, 8, and 9) presented an effective reduction in pain and ODI (≥50% improvement of VAS and ODI compared with pretreatment) at 12 months

The mean pretreatment VAS for LBP decreased from a baseline of 6.5 ± 1.27 to 4.6 ± 1.07 at 1 month (*P* = 0.01), and even further to 4.3 ± 1.63 at 3 months (*P* = 0.02), 3.2 ± 1.40 at 6 months (*P* = 0.004), and 2.9 ± 1.66 at 1 year (*P* = 0.002). The mean ODI score of 42.8 ± 15.03% before treatment declined to 31.2 ± 13.86% at 1 month (*P* = 0.002), 31.7 ± 14.22% at 3 months (*P* = 0.01), 21.3 ± 7.42% at 6 months (*P* = 0.002), and 16.8 ± 9.77% at the 12-month follow-up visit (*P* = 0.002) (Table 3). There were no statistically significant differences between the low-dose group (2 × 10^7^ cells/disc; cases 1, 2, 3, 4, and 5) and the high-dose group (4 × 10^7^ cells/disc; cases 6, 7, 8, 9, and 10) in terms of VAS and ODI scores at each time point. Seven of the 10 patients (cases 2, 4, 5, 6, 7, 8, and 9) showed significant improvement ≥ 50% in the VAS and ODI at 6 months, whereas final treatment success (reduction ≥ 50% in the VAS and ODI compared with pretreatment VAS and ODI) was found in six subjects (cases 2, 4, 5, 7, 8, and 9) at the 12-month follow-up (Table 4, Fig. 2).

**Table 3:** Comparison of patients’ outcomes according to time points

	VAS			ODI		
	Mean	*P* value_WSR	*P* value_paired *t*	Mean	*P* value_WSR	*P* value_paired *t*
Baseline–1 week	0.5	0.4766	0.5212	-10.6	0.0977	0.00489
Baseline–1 month	1.9	0.0098	0.0044	11.6	0.002	0.0014
Baseline–3 months	2.15	0.0156	0.014	11.09	0.0117	0.006
Baseline–6 months	3.3	0.0039	0.0008	21.52	0.002	0.0016
Baseline–9 months	3.4	0.0039	0.0012	22.72	0.002	0.0002
Baseline–12 months	3.6	0.002	0.0003	26.02	0.002	0.0004
Baseline–mean of each visit	2.475	0.0039	0.001	13.725	0.002	0.0018

*VAS* visual analogue scale, *ODI* Oswestry Disability Index, *WSR* Wilcoxon signed-rank test

**Table 4:** Percentage reduction of VAS and ODI at 1, 3, 6, and 12 months post injection and ADC value improvement at 1, 6, and 12 months post injection

Patient number	Reduction in VAS (%)				Reduction in ODI (%)				ADC value			
	1 month	3 months	6 months	12 months	1 month	3 months	6 months	12 months	Pre treatment	1 month	6 months	12 months
1	50	75	63	50	30	40	25	25	1060	1290	1410	1456.7
2	15	15	57	100	41	47	65	91	1423.3	1413.3	1180	1160
3	17	17	17	33	33	27	20	13	1230	1480	1050	1193.3
4	29	0	100	86	36	38	82	82	1056.7	1230	1116.7	1086.7
5	0	25	25	50	19	31	31	75	1623.3	1470	1516.7	1583.3
6	29	29	57	14	17	31	58	36	1036.7	875	ND	ND
7	50	50	50	50	4	15	58	70	1173.3	992.3	1236.7	1063.3
8	33	67	50	50	25	31	59	63	834.3	800.7	998.7	965
9	50	33	50	50	38	25	19	56	1243.2	1366.7	1470	1496.7
10	14	43	29	43	50	48	60	57	1034.6	1226.7	1036.7	1053.3

*VAS* visual analogue scale, *ODI* Oswestry Disability Index, *ADC* apparent diffusion coefficient, *ND* not determined

### Image analysis

**Fig. 3:**
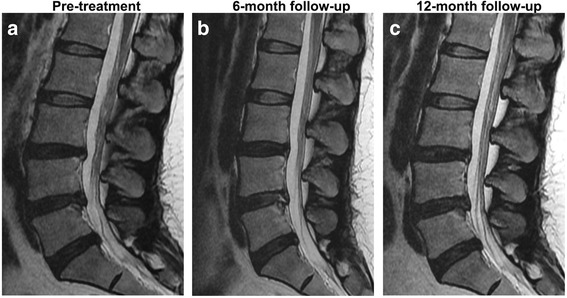
Assessment of disc degeneration grade using T2-weighted MRI. Sagittal T2-weighted images (**a**, pre treatment) show increased signal intensity of the L4/5 intervertebral disc from IV to III at 6 months (**b**) and 12 months (**c**), suggesting an increase of water content (case 1)

Lumbar spine X-ray imaging showed no decrease in the disc height of the transplanted IVD at the final follow-up at 1 year. The severity of disc degeneration was evaluated using the Pfirrmann grading system (grade I, normal to grade V, most severe degeneration with a collapsed disc) based on conventional T2-weighted sagittal images [29]. We then semiquantitatively analyzed changes in water content using ADC mapping from DWI. In all patients, lumbar endplate Modic changes were absent. All patients had Pfirrmann grades of IV at the time of enrollment. There were no cases where degeneration at the injected IVD was worse at the 12-month follow-up. The six patients who achieved treatment success (pain reduction ≥ 50% and ODI improvement ≥ 50%) showed no increase in Pfirrmann grade at the final follow-up. The Pfirrmann grade of the transplanted L4/5 disc increased from grade IV to grade III at the 6-month and final follow-ups in case 1, who achieved significant VAS improvement at 6 months (Fig. 3). Among the six patients (cases 2, 4, 5, 7, 8, and 9) who achieved treatment success at the final follow-up, cases 4, 8, and 9 showed increased water content based on the ADC map at the 12-month follow-up (Table 4). No osteophyte formation or narrowing of the IVD was observed in any case.

## Discussion

MSC-based intradiscal treatment has recently attracted attention for its potential to revolutionize the treatment of chronic discogenic LBP by repopulating the IVD and restoring functional tissue through matrix synthesis by implanted cells and possible beneficial effects on native cells [5]. At least three published clinical studies and one unpublished clinical trial (ClinicalTrials.gov NCT01290367) have used MSC-based therapies to treat chronic discogenic LBP [12, 13, 33] (Table 5). Yoshikawa et al. [13] first reported significant LBP reduction and rehydration of the treated disc following percutaneous injection of autologous BM-MSCs within a collagen sponge in two older patients with markedly degenerated discs. Orozco et al. [12] reported a pilot study of 10 patients with chronic discogenic LBP treated with percutaneous intradiscal injection of autologous BM-MSCs, in which 90% of the subjects reported clinical benefit, and both LBP and disability were greatly reduced at 3 months after transplantation, followed by modest additional improvement at 6 and 12 months. MRI also revealed marginally improved hydration of the treated disc at 12 months [9, 12]. Similarly, Pettine et al. [33] evaluated the use of autologous, nonexpanded concentrated BM cells considered to contain multiple stem and progenitor cells, including MSCs. In total, 26 patients with chronic LBP underwent intradiscal injection of the concentrated BM cells, and significant improvement in the VAS, ODI, and modified Pfirrmann score was found at 3, 6, and 12 months post transplantation. Although the exact mechanism in Pettine et al.’s study remains unclear, patients who received > 2000 colony-forming unit-fibroblasts (CFU-F) per milliliter of bone marrow aspirate showed significantly greater improvement of discogenic LBP compared to patients receiving < 2000 CFU-F/ml [9, 34]. The 36-month results from a 100-patient, four-arm (high dose of 1.2 × 10^7^ mesenchymal precursor cells (MPCs) with HA, low dose of 0.6 × 10^7^ MPCs with HA, saline injection, and HA injection), randomized, placebo-controlled phase 2 trial were recently announced. In that trial, 41% of the low-dose group and 35% of the high-dose group achieved 50% pain reduction at 24 months, whereas 82% of the low-dose group who achieved 50% pain reduction over 24 months maintained treatment success at 36 months (Mesoblast website: file:///C:/Users/user/Downloads/Durable%20Three%20Year%20Outcomes%20in%20Disc%20Disease.pdf) (Table 5).

**Table 5:** Clinical trials using mesenchymal stem cell-based therapies for degenerative disc disease

Year, author	Stem cells	Cell number	Number of patients	Injection	Follow-up (months)	Findings
2010, Yoshikawa et al. [13]	Autologous BM-MSCs	10^5^ cells/ml	2	Intradiscal, single	24	Improvement in pain score and rehydration of the disc in both patients
2011, Orozco et al. [12]	Autologous BM-MSCs	23 ± 5 × 10^6^	10	Intradiscal, single	12	Improvement in pain, disability, and disc hydration
2015, Pettine et al. [33]	Autologous BM concentrated cells	121 (±11) × 10^6^	26	Intradiscal, single or double	12	Improvement in pain scores in patients with higher CFU-F concentrations; improvement on MRI (*n* = 8)
2017, Mesoblast Ltd (unpublished)	Allogeneic MPCs in HA carrier	6 × 10^6^ 1.8 × 10^7^	100	Intradiscal, single	36	Improvement in VAS and ODI in 6 million MPCs injected group
Present study	AT-MSCs in HA carrier	2 × 10^7^ (*n* = 5) 4 × 10^7^ (*n* = 5)	10	Intradiscal, single	12	Improvement in VAS, ODI, SF-36 (*n* = 6); improvement of water content on diffusion MRI (*n* = 3)

*BM-MSC* bone marrow derived mesenchymal stem cell, *MPC* mesenchymal precursor cell, *AT-MSC* adipose tissue derived mesenchymal stem cell, *HA* hyaluronic acid, *VAS* visual analogue scale, *ODI* Oswestry Disability Index, *CFU-F* colony-forming unit-fibroblast, *SF* Short Form, *MRI* magnetic resonance imaging

Despite these positive results showing that MSCs are promising for degenerative disc repair, low cell survival resulting from the hypoxic and inflammatory host environment, cell leakage leading to osteophyte formation, and identification of the optimal cell source and optimal delivery method still present major challenges to MSC therapy [2, 8, 32, 35]. Thus far, it seems that both autologous and allogeneic MSCs can be implanted safely, but immune rejection remains a significant issue [36]. It has been reported that 90% of the transplanted cells leaked out of the degenerated disc following injection in aqueous solution, but this leakage was reduced to 50% with fibrin glue coadministration [2, 35]. Similarly, our previous preclinical study showed that coadministration with Tissuefill® (HA derivative) could enhance the efficacy of intradiscally injected MSCs [32]. The present study differs from previous clinical studies [12, 13, 33] and the four-arm clinical trial (Mesoblast’s clinical trial) described earlier because our patients were coadministered a HA derivative and autologous AT-MSCs rather than bone marrow-derived cells. AT-MSCs can be obtained with a procedure that is less invasive and presents lower risk than harvesting of BM-MSCs. Furthermore, the autologous AT-MSCs in this study showed increased expression of TβRIII, a potential predictor of chondrogenesis. AT-MSCs expressing TβRIII at higher levels are thought to have higher chondrogenic potential through increased susceptibility to TGF-β3-induced chondrogenesis [34]. Although intradiscal injection can cause further injury, leading to more degeneration and cell leakage, current delivery methods are limited to direct injection into the affected discs [37]. To the best of our knowledge, this is the first clinical trial showing the safety and tolerability of combined implantation of autologous AT-MSCs and a HA derivative in patients with chronic discogenic LBP.

The present study was designed as a phase I clinical trial, for which the calculation of adequate sample size is often difficult. Here, we expected that 10 patients would be sufficient to achieve the trial endpoints because the active sample size in phase I clinical trials has been generally reported to be between six and 10 active subjects [38]. Using this sample size and study design, we were able to obtain the following primary findings: combined implantation of AT-MSCs and the HA derivative is safe and tolerable for treatment of chronic discogenic LBP; additionally, at 6 months following AT-MSC transplantation, seven patients (cases 2, 4, 5, 6, 7, 8, and 9) showed significant reduction of VAS, ODI, and SF-36, whereas at 12 months only six patients (case 2, 4, 5, 7, 8, and 9) showed significant improvement of pain and disability. The treatment success rate was not different between the low-dose (2 × 10^7^ cells/disc) and high-dose (4 × 10^7^ cells/disc) groups, and among the six subjects (cases 2, 4, 5, 7, 8, and 9) who achieved treatment success, three (cases 4, 8, and 9) were determined to have increased water content based on the increased ADC value determined from diffusion MRI. We used the ADC values to assess the water content in the degenerated disc because ADC values have been reported to be a more reliable method of assessing subtle changes in water content compared with T2-weighted imaging and T2 mapping [39] (Table 4).

The exact mechanism by which the combined implantation of AT-MSCs and the HA derivative led to improvement of chronic discogenic LBP in the present study remains unclear. Based on our previous preclinical study, however, we assume that injection of MSCs into the degenerated disc improves ECM production by the degenerated host NP cells, increases NP-like gene expression, and modulates the immunological response of NP cells to inflammatory cytokines. The immunomodulatory effects of MSCs on NP cells within the degenerated disc could potentially inhibit the inflammatory cascade, thereby preventing ingrowth of pain-inducing vasculature and nerve fibers [2, 8, 32]. Additionally, suspension of the cells in HA derivative for coadministration may prevent cell leakage, reduce the risk of uncontrolled differentiation of MSCs into osteoblasts, and enhance cell attachment and cell survival [32].

**Fig. 4:**
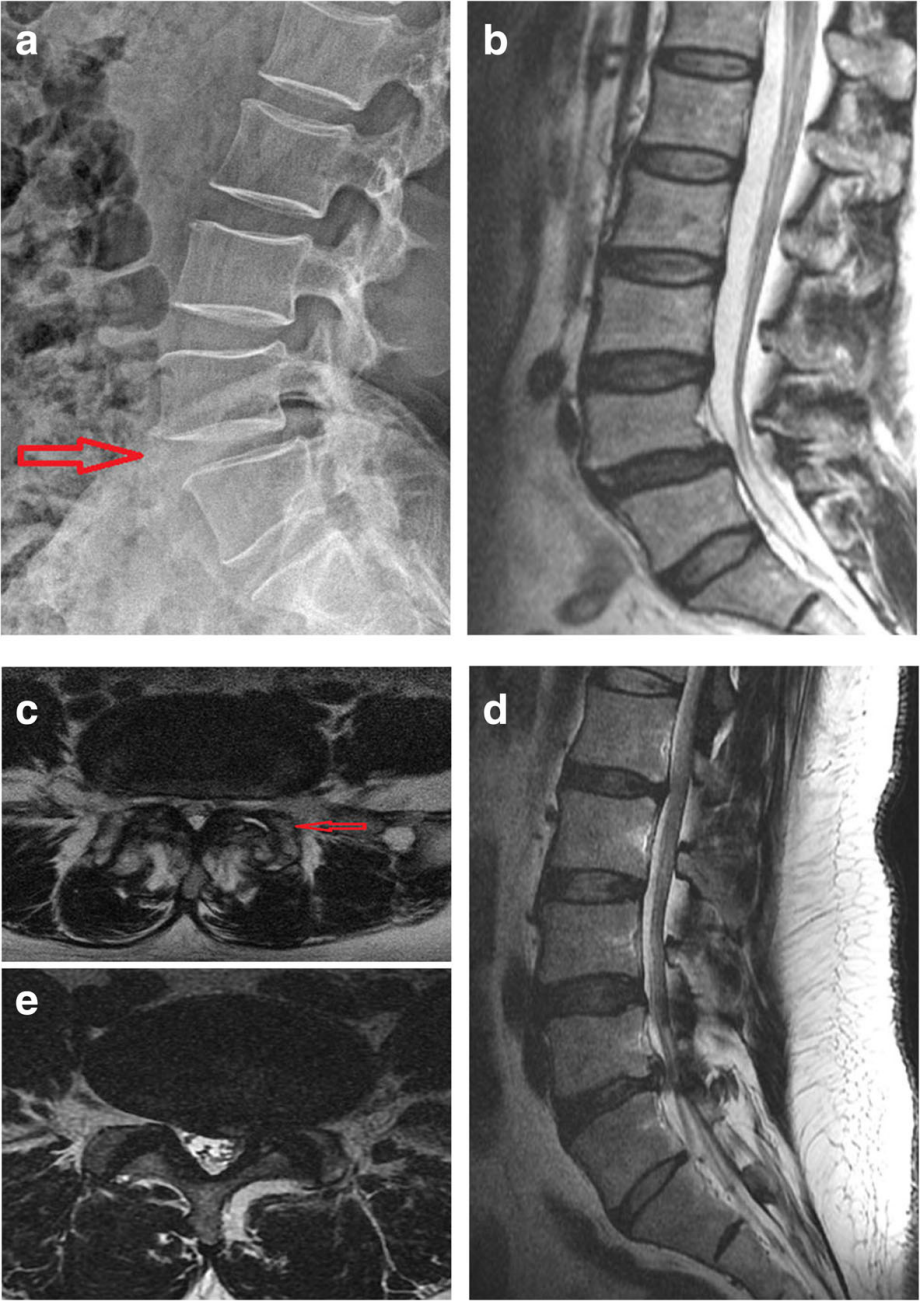
Possible causes of treatment failures in cases 3 and 6. Lumbar lateral X-ray imaging (**a**), T2 sagittal MRI (**b**), and T2 axial MRI (**c**) of case 3 showing degenerative spondylolisthesis of L4 on L5 (slippage or displacement of L4 vertebra compared to L5 vertebra) and spinal stenosis. T2 sagittal MRI (**d**) and T2 axial MRI (**e**) of case 9 revealed left-sided L4–5 herniated NP with decreased disc height

We also evaluated the potential causes of treatment failure (pain reduction < 30% or ODI improvement < 30%) in four patients (cases 1, 3, 6, and 10). In case 1, the patient was overweight and reported significant pain relief for LBP (50% pain relief) at the 12-month follow-up, but the ODI improvement was < 30%. Although the patient was not classified in the treatment success group, notable increases in Pfirrmann grade and in the ADC value were found at the 6-month follow-up (Fig. 3). Initially, case 3 complained of LBP and pain in both buttocks. Initial lumbar X-ray imaging and MRI showed degenerative spondylolisthesis (forward displacement of L4 on L5), facet joint arthritis, and spinal stenosis at the L4/L5 level; flexion and extension radiography showed no lumbar instability (Fig. 4). The patient had no neurogenic intermittent claudication or radiculopathy, which was supported by electromyography and a nerve conduction study. The pain did not improve even after medial branch nerve blocks, and the patient was included in this study based on discographic findings. Case 6 was obese (38 kg/m^2^) and had a disc protruding to the left side at the L5/S1 level, with a disc height reduction of approximately 21.4%. Case 10 had depressive symptoms (Beck Depression Inventory initial score 14), which might have resulted in treatment failure. Although all patients were enrolled after lumbar medial branch block and discography to rule out other causes of LBP, the LBP was still not successfully eliminated in cases 6 and 10, possibly because of other potential confounding factors for chronic LBP such as obesity (case 6) and depression (case 10). In addition, other structural etiologies for chronic LBP that could have prevented treatment success here may include spondylolithesis (case 3), spinal stenosis (case 3), facet joint arthritis (case 3), decreased disc height (case 6), and disc herniation (case 6) (Fig. 4).

Thus, careful patient selection is essential for achieving therapeutic success in stem cell therapy for chronic discogenic LBP because MSC-based therapies should not be thought of as a cure-all for spinal pain [40]. Given the multifactorial nature of chronic LBP, it is difficult to isolate patients in which IVD degeneration is the only contributor to LBP without a larger-scale study, but the current study supports the safety and tolerability of such a clinical trial. Moderate IVD degeneration has been considered an ideal target for stem cell therapy. Patients with nondiscogenic LBP and those with advanced disc degeneration or severe annular compromise may not be ideal candidates for this therapy [40]. Furthermore, it is necessary to consider the ideal cell dose to administer. The present study showed no significant difference between the low cell dose and high cell dose, suggesting that an even lower cell dose may still be efficacious. A low cell dose might actually be beneficial due to the poor nutritional supply of the NP, which could result an inability to support higher numbers of transplanted cells. In such a case, a high cell dose that exceeds the limit for donor cell viability in the NP environment could lead to deleterious accumulation of dead cells and waste products [2, 8]. Other important considerations could be the duration of cell survival after implantation and the degradability of the scaffold.

Our study is a single-arm, open-label, phase I pilot study, and thus caution should be applied when drawing any conclusions regarding long-term safety and efficacy. Although there was no placebo group in this pilot study, a placebo effect is unlikely because coadministration of AT-MSCs and HA derivative provided significant improvement of patient outcomes (VAS/ODI and MRI improvement), and both the patients and attending physician were blinded to the treatments that each patient received. Large-scale clinical trials assessing the optimal cell source, cell dose, scaffold, and relevant clinical endpoints are needed to define the true pathology that will benefit from stem cell therapy and the appropriate therapeutic regimen. However, we propose that coadministration of AT-MSCs and the HA derivative may provide a safe and tolerable treatment for chronic discogenic LBP resulting from moderate IVD degeneration.

## Conclusions

Our clinical study confirmed the safety and tolerability of coinjection of AT-MSCs and a HA derivative in patients with IVD degeneration. Significant improvements in the VAS pain score and ODI score were demonstrated in six of 10 patients. The rehydration of the discs in three of six patients according to ADC mapping in conjunction with sustained pain relief throughout the 12-month duration of the study demonstrates the promise of this regenerative medicine approach. These favorable results support the initiation of phase II, prospective randomized clinical trials.

## Abbreviations

AE: Adverse event
AT-MSC: Adipose tissue-derived mesenchymal stem cell
BMI: Body mass index
CT: Computed tomography
DWI: Diffusion-weighted image
ECM: Extracellular matrix
HA: Hyaluronic acid
IVD: Intervertebral disc
LBP: Low back pain
MPC: Mesenchymal precursor cell
MRI: Magnetic resonance imaging
MSC: Mesenchymal stem cell
NP: Nucleus pulposus
ODI: Oswestry Disability Index
SAE: Severe adverse event
SF-36: Short Form-36
TE: Echo time
TI: Inverse time
TR: Repetition time
TβRIII: Type III TGF-β receptor
VAS: Visual analogue scale
